# The American Medical Association

**Published:** 1863

**Authors:** 


### THE AMERICAN MEDICAL ASSOCIATION.

The Fourteenth Annual Session of the American Medical Association convened in Bryan Hall on Tuesday, June 2. There was a full attendance, most of the States being represented.

#### ASSEMBLING OF THE CONVENTION.

The Convention was called to order at 11 o'clock by Wilson Jewell, of Pennsylvania, the first Vice-President of the Association during the last three years. The remaining retiring officers occupied seats on the platform, with the exception of the President, who is deceased. The following are the gentlemen who have held office since the year 1860:—

*President.*—Eli Ives, Connecticut.

*Vice-Presidents.*—Wilson Jewell, Pennsylvania; A. B. Palmer, Michigan; R. D. Arnold, Georgia; Joseph N. McDowell, Missouri.

*Secretaries.*—S. G. Hubbard, Connecticut; H. A. Johnson, Illinois.

*Treasurer.*—Casper Wister, Pennsylvania.

Rev. R. L. Collier, pastor of the Wabash Avenue Methodist Episcopal Church then invoked the Divine blessing upon the deliberations of the Association.

#### ADDRESS OF WELCOME.

The Address of Welcome to the Delegates was made by Dr. N. S. Davis, on behalf of the physicians of the City of Chicago and the State of Illinois.

It became his pleasing duty, on behalf of the profession of medicine in Chicago, as well as Illinois, he said, to welcome them, which he did with great pleasure. This was a new city, and, as a consequence, destitute of many of those attractions to be met with in other and older cities. Yet all the institutions of the East were here in their incipiency, and were flourishing. This city has grown in thirty years from an Indian tract to a population of one hundred and forty thousand people, and to a well-organized and highly-educated community. The public schools, academy of sciences, universities, colleges, historical society, and commercial structures, all proved how rapidly Chicago has progressed. They were welcomed, however, not to these, but to the hearts and homes of the citizens,—doubly welcome on account of the three years that had elapsed since their last meeting. The interruption was made from year to year, in the hope that they would again be able to meet from all sections of the country and extend the common hand of fellowship to all their brethren. But it was necessary to retain their organization, even though their brethren did not join in the convention. Nevertheless, he did look forward to the time when they could all again assemble under one flag, with one nationality, to resume their mutual researches into the secrets of that philosophy which bore so intimate a relation to the welfare of the whole family of man.

They were doubly welcome, because their society was not based on a selfish aim,—its end was to advance the educational and scientific interest of a profession whose aim and province it is to gather all the knowledge which tends to alleviate human pain, prolong human life, and perpetuate human happiness. For this great object they had travelled thousands of miles. The prairies in this State were broad, but not more open than their hearts, and, if they failed in doing all that could be wished for their comfort, it would be on account of the crowded condition of the city. He concluded by saying, that they had gathered as friends, and he believed that their business would be transacted in harmony and peace, and that when they adjourned it would be with the feeling that it was good to be here.

They were assembled in the midst of great national excitement, but their business would be transacted thoroughly and satisfactorily, because it did not conflict with the interests of any class, —it ministered to all. In the profession they were all patriots, all lovers of their country; and if, in their deliberations, they could bring out but one fact which would tend to alleviate the sufferings of those who were fighting for their country, they would be amply repaid. He again bid them doubly welcome.

REPORT OF THE COMMITTEE OF ARRANGEMENTS.

The Report of the Committee of Arrangements was then read by Dr. Davis. It contained an allusion to the unsettled state of the country, as the reason why the Annual Meeting had been postponed for two years. The last was held in 1860, in New Haven, Connecticut. In the following year,—1861,— the session was postponed on account of the wish that both sections of the country should, as usual, be represented, and it was hoped that before the next year the war would be ended. At the time when the next meeting fell due, many of the profession were absent in the hospitals. It was decided by the New York State Association last winter that a convention should be held this year; and, whether the action was judicious or not, it was indorsed by every society so far as heard from; and every journal in the States, except one, had spoken favorably of the movement.

It had been found impossible to secure aid from the railroad corporations, as they had made mutual agreement not to grant passes to conventions, and the Canal Convention was an exception to that rule. The attendance of delegates and prominent members was large, and indicated a satisfactory and profitable meeting. He then directed the attention of the members to the cards of invitation to the evening meetings, and requested their attendance. The Report was adopted.

NAMES OF DELEGATES IN ATTENDANCE.

The Roll of Members was then called by the Secretary. The following are the names of those present:—

*Vermont.*—J. N. Stiles, Lewis Emmons.

*Massachusetts.*—Henry Cutter, Appleton Thorne, Edward Barton, James P. Lynde, Ebenezer Stone, J. P. Kendall, Benj. E. Cotting, John Homans, John C. Dalton, M. D. Southwick, E. P. Abbe, John Green.

*New York.*—Henry G. Davis, Guido Furman, Alden March, Daniel P. Bissel, James S. Whaley, Thomas C. Brinsmade, J. S. Sprague, C. C. F. Fay, Edward Storck, E. S. F. Arnold, E. W. Cheeney, W. N. Blakeman, Howard Townsend, H. Nicholl, E. Tobie, H. S. Downs, C. C. Wyckoff, Alf. Underhill, J. H. Griscom, L. B Cotes, Julius Homberger, C. W. Harvey, James McNaughton, Daniel Holmes.

*Connecticut.*—Stephen G. Hubbard, L. N. Beardsley, B. H. Catlin, A. W. Barrows.

*New Jersey.*—Wm. Pierson, Jr., D. M. Sayre, John Blain, Isaac S. Cramer.

*Delaware.*—H. F. Askew, James Couper.

*Pennsylvania.*—Wilson Jewell, Wm. Mayberry, Edward Wallace, B. Richardson, John R. Thomas, E. H. Mason. Wm. L. Richardson, T. N. Troth.

*Virginia.*—J. C. Hupp.

*Ohio.*—W. S. Battles, J. M. Taggart, W. W. Jones, A. H. Agard, K. G. Thomas, S. O. Almy, L. M. Lawson, W. B. Davis.

*Indiana.*—B. S. Woodworth, A. M. Vickery, A. J. Erwin, A. P. Ferris, L. D. Personett, James Ferris, S. A. Freeman, L. D. Glazebrook, James F. Hibbard.

*Michigan.*—A. B. Palmer, E. A. Egerry, H. O. Hitchcock, S. D. Richardson, Lewis Davenport.

*Illinois.*—E. L. Holmes, Geo. K. Amerman, Edmund Andrews, John Ten Broek, C. R. Parks, T. D. Fisher, Geo. W. Hall, David Prince, E. Andrews, N. Wright, W. O. Chamberlain, R. Rouse, E. A. Steele, A. Fisher, M. J. Johnson, J. H. Hollister, D. Pierson, M. F. Dewitt, S. Wickersham, J. P. Ross, Henry Wing, Chas. Gorham, S. W. Noble, Ira Hatch, T. F. Worrell, F. B. Haller, H. A. Johnson, R. Spitler, G. Paoli, H. Noble, D. M. Tucker, Orrin Smith, A. J. Crain, T. K. Edmiston, J. J. Lake.

*Wisconsin.*—Chas. L. Stoddert, H. Adams, Harmon Van Dusen, E. S. Carr, D. Wilber.

*Iowa.*—J. W. H. Baker, Samuel C. Lay, Jos. Sprague, D. L. McGugin.

*Kansas.*—D. W. Stormont, C. A. Logan.

*Tennessee.*—W. K. Boling.

*Army and Navy.*—C. C. Cox, J. Simpson, A. R. Terry, John B. Porter, Benj. Palmer, M. K. Taylor, Ralph N. Isham.

The Secretary announced that in regard to returning members of this Association to their homes, the Secretary of the Canal Convention had signified that they were at liberty to become recognised as delegates to the Canal Convention by registering their names with him.

COMMITTEE ON NOMINATIONS.

The delegates from the several States then resolved themselves into sub-committees, and appointed their representatives on the Committee for Nomination of officers, as follows:—

*Vermont.*—J. N. Stiles.

*Massachusetts.*—John Homans.

*Connecticut.*—L. N. Beardsley.

*New York.*—Jos. McNaughton.

*New Jersey.*—John Blain.

*Delaware.*—H. F. Askew.

*Ohio.*—W. S. Battles.

*Indiana.*—James F. Hibbard.

*Pennsylvania.*—Wm. Mayberry.

*Michigan.*—H. O. Hitchcock.

*Kansas.*—D. W. Stormont.

*Virginia.*—John C. Hupp.

*Iowa.*—J. H. W. Baker.

*Wisconsin.*—H. Van Dusen.

*Illinois.*—H. Noble.

*Tennessee.*—W. K. Boling.

*Maryland.*—Dr. Cox.

*The Army.*—Joshua Simmons.

VALEDICTORY ADDRESS BY THE ACTING-PRESIDENT.

Dr. Wilson Jewell, Acting-President of the Association, then delivered his Valedictory Address. Since their last meeting, he remarked, the most tremendous events had transpired; the country had been plunged into civil war, and the best government that ever existed on the face of the earth had trembled as on the brink of dissolution. It was not strange if the troublous element had found its way into their counsels, yet he had hope still that the present struggle would be gloriously ended by a restoration of the Union- The cause was based on the eternal principles of civil and religious liberty, and could not fail.

The speaker then turned to the subject which was most intimately connected with the objects of the Convention, and spoke of the noble part taken in the struggle by the devoted members of the professson, who, amid the thunders of battle and the din of arms, worked firm and self-possessed to mitigate the horrors of the strife, and risked being killed or taken prisoners of war rather than desert the path of duty. Theirs' was no warrior's ambition; they were stimulated by no wish, save that of alleviating human suffering. Many of their members were in the army, and some slept the sleep of death. First, and highly valued among them, was their respected President, Eli Ives, whose knowledge and experience had rendered him so valuable a medical practitioner, and whose private virtues endeared him to all. The future usefulness of the Association was one of the great aims of their late President, and he predicted great things of its future.

The orator then took a retrospective view of the progress of the Association, and spoke of the signs, in the present condition and standing, which point to a bright and influential future. He quoted passages from many eminent medical men, in which the future of the Association was spoken of and hints for improvement given. He would, however, direct their attention to another subject, not that he loved Cæsar less, but Rome more. He would speak of Hygiene, a science which bears no modern date, but claims its origin in the antedelluvian age, is now so little understood, and presents an illimitable field for research. The fearful responsibilities of their calling should stimulate them to a thorough course of study in all that pertains to the preservation of health, the extension of the term of existence, and the alleviation of disease where prevention rendered impossible. There was room to hope that the American Medical Association would throw out a light, which, in the medical world, would equal the refulgence of that bright ray which shone out from the retreat of the Wittenburg student and dissipated the darkness which until then brooded over the theological firmament. Yet this illustration could ensue only upon a careful study of the laws of Hygiene. Not that the subject was incompatible with the design of the Association; it had done great service in that department, and time would fail to tell the aid rendered by it in the past. But the vestibule of the Hygienic temple alone had as yet been attained. The method of curing disease had, heretofore, attracted not too much of attention, but it had, perhaps, thrown into the background those sanitary considerations which will teach how to prevent disease by conformity to the laws of health. This was a reform much needed, but there seemed to be a perpetual obstruction presented to its progress, and a private prejudice in the popular mind against it. He would propose a two-fold method whereby the evil might be remedied. It was to elevate Hygiene as a branch of scientific study, and give it a distinct chair in the medical colleges. He would constitute it as a cirriculum of study which was essential to the reception of a diploma. He counseled also the adoption of some more popular and successful plan than had otherwise been pursued, for enlightening the public mind on the relations of preventive measures to the health of the people. The etiology of disease was the basis of the science of preventive medicine. This being better understood than formerly, and one of the good fruits springing out of the present struggle would be the elimination of a multitude of facts bearing on the relations of military discipline to military health, and the consequent efficiency of the soldier. These facts were of incalculable value, and would exercise a largely beneficial influence upon the health of future ages. Already a work of this kind had been authorized by the general government, which will contain an elaborate classification of military diseases and the influence of hygienic regulations thereupon. The time was, probably, not far distant when each State would have its bureau of health, and recognize the indissoluable relation existing between sanitary conditions and moral developments, as well as on the physical organism. Already two cities of the Union had taken vigorous action in this matter, and the Garden City might well claim the credit of having set the example of aiming to insure civic healthfulness. He recommended that the word "Hygiene" should be written in letters of gold on the escutcheon of the Association.

On motion of Dr. Sprague, of New York, the thanks of the Convention were given to the retiring President for his able, eloquent, patriotic, and scientific address, and a copy was requested for publication.

### CANDIDATES FOR PERMANENT MEMBERSHIP.

The Committee of Arrangements then reported the following as names of gentlemen who were candidates for permanent membership, and recommended their admission:—

Walter Hay, Thomas Bevan, John McAllister, John Bartlett, M. O. Heydock, Niel P. Peterson, R. C. Hamill, H. N. Hurlbut, .V L. Hurlbut, and H. Webster Jones, of Chicago; E. C. Lardner, of Vermont; S. W. Bicknell, Beloit; E. W. Jenks, of Sturges; Henry Durham, LaSalle: Silas Earle, Onarga; and W. W. Sedgewick, of Sandwich. The candidates were unanimously elected.

The following were then elected as members by invitation:—

A. L. Merriam, Sandwich, Ill.; L. D. Glazebrook, St. Pierre, Ind.

The President then appointed Drs. Pearson, Beardsley, and Cutler to draft resolutions expressive of the sense of the Association respecting the death of the late President.

The Convention then adjourned till 3 o'clock.

AFTERNOON SESSION.

The Convention reassembled at 3 o'clock, and the Committee on Nominations was called to make their Report. They recommended the following

FOR OFFICERS:—

*President.*—Dr. ALDEN MARCH, of New York.

*Vice-Presidents.*—Drs. JAMES COUPER, of Delaware; DAVID PRINCE, of Illinois; C. C. COX, of Maryland; and E. S. CARR, of Wisconsin.

*Secretaries.*—[Not to be appointed until the place of the next meeting is known.]

*Treasurer.*—Dr. CASPAR WISTAR, of Philadelphia.

REPORT OF THE TREASURER.

The Report of the Treasurer was read by Dr. Askew, of Delaware, the Treasurer being unable to attend. He reported that, owing to the unsettled state of the country and the advanced price of printing, it would be necessary to print only such papers as were of great value, and to condense those as much as possible, or the treasury could not bear the cost. The proceeds of volumes sold were $1,982 25. Balance on hand last year, $597 61; balance on hand this year, $504 21. The Report was adopted.

The Committee on Publication reported the result of their labors during the year, and the number of volumes now in their possession. The Report was accepted.

AN UNUSUAL CASE.

Dr. Griscom, of New York, reported a very interesting case of diarrhœa adiposa which occurred in the New York Hospital, under his notice; it was cured by the free use of whiskey and porter, but, on being placed in the House of Detention, and thus debarred from its use, the symptoms returned. The speaker said that the case was a very unusual one, not more than twenty-six cases having ever been reported. He considered that the commonly received opinion, that abnormal acretions of oleaginous substance arose from pancreatic secretion was unfounded.

### REPORT ON PRIZE ESSAYS.

The Report of the Committee on Prize Essays was read by Dr. D. L. McGugin, of Iowa. He reported that only one essay had been received, which is worthy of the prize medal. It is an inquiry into the properties and physiological uses of Veratrum Viride, with notices of its alkaloid, Veratria, as derived by certain processes. He considered the essay as worthy of publication and of the prize.

Dr. Lawson moved that the prize be awarded to the author, and the paper forwarded to the Committee on Publication.

Dr. Cox moved that the essay be referred to a special committee, of which Dr. McGugin shall be chairman, and read the essay, and report at some future time.

The motion of Dr. Lawson prevailed, and the name of the author was then announced,—Samuel R. Percy, M.D., Professor of *Materia Medica* and Therapeutics in the New York Medical College. The announcement was received with cheers. The prize is one hundred dollars.

The Report of the Committee on Nominations was made and accepted. The nominations were accepted, and the gentlemen were conducted to their places on the platform by a delegation of two to each appointed by the Chairman. The newly-inducted President briefly returned thanks for the honor conferred.

### REPORTS OF SPECIAL COMMITTEES.

Reports of Special Committees were then called for. A communication was received by Dr. Davis from Dr. E. R. Squibbs, of New York, Chairman of the Committee on "the practical workings of the United States Law relating to the inspection of drugs and medicines," stating that he could not attend, and offering to report next year. Agreed to.

Several other gentlemen presented in person the request to be allowed to continue, with the same result.

Dr. A. K. Gardner, of New York, presented a paper on the use and abuse of pessaries, but the reading was postponed till this morning.

No changes were made in any of the committees, and they were all continued for another year.

THE HUNTER MEMORIAL.

The Committee on the Hunter Memorial reported that the sum of $357 had been raised, in one dollar subscriptions, towards the Hunter fund, a portion of which had been forwarded to London. The smallness of the contribution was imputed mainly to the fact that the monument would stand on British soil, and the indifference felt by England about the present national trial had checked the enthusiasm. The Report was contained in a letter from Dr. Bowditch, which was received and placed on file. It was also decided that the account be closed and the balance retained by Dr. Bowditch.

NEW MEMBERS.

The Committee of Arrangements proposed the following gentlemen to be elected as permanent members:—

Drs. Daniel B. Brengle, Manchester; Van Courtland Secord, Galena; J. B. Samuel, Carrolton; David Dodge, Chicago; James S. King, Lemont; D. F. Crouse, Mount Carroll; all of Illinois. The nominations were confirmed.

THF USE OF MERCURY BY THE ARMY-SURGEONS.

Dr. Lawson called attention to the recent order of the Surgeon-General prohibiting the use of mercurials and tartarized antimony by the army Surgical Corps. He moved that the Society express its disapprobation of the order. The subject was referred to a committee, with instructions to inquire into the facts and report, the committee to consist of one member from each State.

MEDICAL PROVISION FOR RAILROAD ACCIDENTS.

Remarks were then made by Dr. Arnold, of New York, on the necessity of making medical provision for railroad accidents. He distributed printed copies of papers read by him before the State Medical Society and the Academy of Medicine, both of New York.

ARMY-SURGEONS.

Dr. Cox called attention to the want of a recognition of Army-Surgeons, and urged that relative rank should be accorded to them. At present it was not possible for a Surgeon to rise above the rank of Major. He, therefore, offered the following Resolutions:—

*Resolved,* That a Committee of five be appointed by the chair to draft a memorial to Congress asking the enactment of a law by which Surgeons in the service of the United States Army may be accorded relative rank in the same.

*Resolved,* That each medical gentleman present be urgently invited to use every proper influence with the Members of Congress from his respective district, to urge the passage of a law, favorable to the object, at the ensuing session of Congress.

The resolutions were seconded by Dr. McGugin in an able speech, in which he reviewed the relative responsibilities of the Surgeon and Commander, and spoke of the injustice perpetrated in the case of the former. The resolutions were discussed by several other delegates, and were, finally, adopted.

The Convention then adjourned till 9 o'clock A.M.

---

WEDNESDAY MORNING SESSION.

The Minutes of the previous sessions were read and approved.

A large number of additional members from several States were announced as having arrived and registered their names as delegates, including a large number of the physicians and surgeons of this city.

The following gentlemen were admitted as members of the Association, by invitation:—

Dr. J. H. Foster, Libertyville, Ill.; W. G. Millar, Rockford, Ill.; J. A. Brown, Kankakee City, Ill.

The following permanent members of the Association were elected:—

Tiffin Sinks, Leavenworth, Kansas; W. C. Hall, Fayetteville, O.; Hiram Wanzer, Chicago, Ill.; H. K. Dean, Maunkport, Ind.; H. C. Robbins, Newark, Ill.; E. J. Duffield, Woodstock, Ill.; W. Jaynes, Yankton, Dakota Ter.; C. M. Clark, Galva, Ill.

The Reports of Committees being in order, on motion, that of the Committee on Medical Education was postponed until the afternoon session.

The Committee on Appointments made their report, which, on motion, was accepted. Pending its adoption, it proposing Baltimore, Maryland, as the next place of meeting, considerable discussion arose, various members proposing different places. The member from Maryland advocated the feasibility of appointing the next meeting at Baltimore, as a national measure. It is for the interest of the Association and the country to hold the meeting *as far South as possible.* The effect of holding it at Baltimore would be a healthy one upon that city and its medical interests. Men of wealth and influence would open their doors and extend warm hospitality to the members of the Association. The question finally resolving itself into a choice between Baltimore and New York City, the latter was unanimously voted for as the place for holding the next meeting.

The balance of the Report, concerning the officers of the next meeting, committees, etc., was referred back to the Committee for reconstruction, rendered necessary by the substitution of New York for Baltimore.

On motion, a committee, consisting of one member from each State, was appointed to investigate and report upon the present and a better ambulance system in the Army of the United States.

A resolution of thanks to Dr. Wilson Jewell, late Acting-President, for the able and dignified manner in which he has presided over the deliberations of the Association was unanimously adopted.

A resolution, requiring the appointment of a committee to urge the compulsory vaccination of every person in the United States, was referred to the Section on Hygiene.

The Report of Dr. A. K. Gardner, of New York, regarding the use and abuse of pessaries, the reading of which was yesterday postponed until this morning, was called up, as next in order, and, on motion, the reading of it postponed until next year.

The Committee appointed to prepare suitable resolutions appropriate to the loss of the Association by the death of its late President, the late Dr. Eli Ives, of Connecticut, made their Report, which, after a slight amendment, was adopted.

The Committee on Voluntary Communications presented an abstract of a paper by Dr. Andrews, of Chicago, on "Diathesis,—their surgical relations," which was read by the author. Approved, and referred to the Committee of Publication.

This paper of Dr. Andrews called up Dr. Hibbard, of Indiana, who combatted, in a rather lengthy speech, some of the principal features presented.

Dr. Andrews replied, in support of his arguments and statements, developing, from his experience, the truth of the position which he assumed.

Other members participated in the discussion.

The meetings of Sections having been abolished, the President appointed as the Committee on Compulsory Vaccination, which had previously been referred to the Section on Hygiene, Drs. Hibbard, of Indiana, Jewell, of Pennsylvania, and Griscom, of New York.

The meeting then adjourned until afternoon.

AFTERNOON SESSION.

According to a resolution passed this morning, Dr. D. J. Macgowan, of New York, from China and Japan, was invited to address the Association. He explained to the meeting the professional bearings of his proposed scientific and industrial expedition to the unknown parts of Eastern Asia. Investigation in relation to the history of epidemics, in the *Materia Medica*, and into the ethnology of those lands, cannot fail to elicit many facts which promise to be of incalculable value to medicine and the collateral sciences. Dr. M. further expressed a hope that the Association would take some measures to induce the Haytien Government to undertake the acclimatization of Cinchona trees, (quinine plants.) He gave an account of the success of the Dutch in Java, and of the English in India, and fully believes that in St. Domingo these invaluable plants might be readily cultivated, and thus secure additional supplies of this great remedy in fevers.

Dr. Macgowan has been in correspondence with the Haytien Ambassador in Washington on the subject, and solicits the influence of the profession in urging the institution of the necessary experiments in those portions of America, north of the equator where the soil and climate seem to afford sufficient encouragement.

In the course of his remarks, the speaker gave an account of the standing of the medical profession in China and Japan, of their medical literature, &c., also stated the remarkable fact that they had made many discoveries in the use of remedies for certain diseases, in some cases either actually the same or very similar to those discovered and used here.

The Chairman of the Committee of Arrangements announced the following names of physicians who were elected permanent members:—

J. B. Buchtell, South Bend, Ind.; C. Truesdale, Rock Island, Illinois; L. F. Warner, and M. Parker, of Chicago. Drs. L. T. Hewins, Oak Alla, Wis., and C. J. Taggart, Beloit, were elected members by invitation.

Dr. C. C. Cox, from the Committee on Medical Education, read an able scientific paper on the subject, reviewing the past history of the profession in this respect, and the absence of proper attention to the subject. Many valuable suggestions, as to needed improvements, were also made. After the rendering of this Report, the Committee submitted the following resolutions, which, after discussion, were adopted:—

*Resolved*, That a preliminary education in English, Latin, Mathematics, and Physics, constitute an essential pre-requisite to the admission of a student of medicine into the office of a medical preceptor, or as a matriculant of a respectable medical college.

*Resolved*, That the advancement of medical education demands a more extended and symmetrical course of instruction in the colleges, and a more thorough and impartial examination for the degree of Doctor of Medicine than at present prevails.

*Resolved*, That Medical Jurisprudence and Hygiene are highly important branches of Medical Science, deserving the careful consideration of all medical teachers and schools.

*Resolved*, That societies for medical improvement,—State, district, and county,—are important auxiliaries to the advancement and promotion of science, and are, therefore, highly recommended by this body, as valuable levers in the cause of medical education.

The Committee appointed to make a report upon the recent order of the Surgeon-General, prohibiting the use of mercurials and tartarized antimony by the Army Surgical Corps, made a majority report through Dr. Lawson, of Cincinnati, and an entirely antagonistic minority report by Dr. Woodworth, of Indiana. The former strongly favored the use of these remedial agents in the Army, and the latter as strongly discountenanced their use there. Each report was backed by resolutions rigidly endorsing the language of the report; after an animated discussion, the Association adjourned.

---

THURSDAY MORNING SESSION.

The Association convened at 9 o'clock. After a partial reading of the minutes, the further reading was dispensed with.

The following gentlemen were admitted members by invitation:—

Isaac Snyder, Jackson, Mich.; R. B. Treat, Janesville, Wis.

The following were admitted as permanent members:—

Granville S. Thomas, Joliet, Ill.; J. S. Pashley, Osceola, Ill.

Dr. Cox, of the army, announced the sudden departure of Dr. Wilson Jewell, of Pennsylvania, caused by receiving intelligence of the unexpected death of a son, and offered a resolution of condolence, which was adopted.

Regular business being in order, the reports of Committees were taken up.

Dr. Gilbert, of the army, in behalf of the Committee on the extinction of the Aboriginal Races, reported progress; and, on motion, the Committee was continued another year.

The President having announced that the order of the Surgeon-General, U. S. A., debarring calomel and tartar emetic from the use of army-surgeons, and which was previously referred to a Committee, was in order; by consent of the Association the Committee on the subject offered a substitute for the resolutions introduced yesterday.

Pending the discussion, previous to the vote, Dr. Cox, of the army, said, substantially as follows:—

While the Association had the right to protest against the order of the Surgeon-General, he wished it to remember that the order referred, exclusively, to the corps of army-surgeons under his control, and had no reference to the use of those drugs in private practice. The order originated in the abuse of Calomel by a number of incompetent surgeons in the army, appointed by the Governors of the several States, who consider the *liver* the pack-horse of the human system. The Medical Bureau of the United States comprises men of science, who understand how far the evil has been perpetuated and the necessity of correcting its abuses. The fact that other mercurials have not been interfered with, shows how great the necessity that exists for an order so apparently sweeping, and which the Association deems so arbitrary.

He did not desire to protract the debate, but felt it due his position to say something before the final vote should be taken. He was not up either to defend or condemn the order. In a long practice he had seen the abuse of calomel in improper hands, as well as its benefits from its legitimate and judicious use. He wished a discrimination to be made between the propriety of the order and the motives of the Surgeon-General. That gentleman's high character and motives are not to be questioned in this or any other public body. He deserved the thanks of the profession for the wholesome interest he had taken in the subject.

Dr. Cox's position called up several members in reply. Calomel had fallen under the ban of an "unwise, unnecessary, and unprofessional order," and that order received animadversion, ridicule, and unstinted opposition. The discussion became general, and while some desired to place no obstacles in the field, their opinion of the order was of a character that culminated in the following resolutions, which were adopted:—

*Resolved*, 1.—That this Association condemn, as unwise and unnecessary, the circular of the Surgeon-General prohibiting the further supply of Calomel and Tartar Emetic for use in the Army; and that we regard such an order as an indignity to the military surgeons, while it is in direct opposition to the opinions of the regular profession of medicine.

*Resolved*, 2.—That the withholding ordinary medicines from the army-surgeons implies a want of confidence in their skill as a body, which, if true, calls for the prompt interposition of the proper authorities; but if the imputation of a want of skill is unfounded, as we believe it is, the refusal to supply proper medicines is wholly unjustifiable.

*Resolved*, 3.—That Circular No. 6 being impolitic and prejudicial to the interests of the service, it is the decided sense of this Association, that a due regard for the welfare of the Army requires, and we do, therefore, earnestly recommend, the recision of that Circular, and the substitution of the more just and philosophical method of correcting abuses, if any exist, by holding each surgeon, individually, responsible for the proper discharge of his appropriate duties.

The entire report, giving a history and details of the subject, in the same spirit, was also adopted.

On motion, it was resolved that a copy of the above resolutions be forwarded to the President of the United States, the Surgeon-General, of the United States Army, and the Secretary of War.

The Nominating Committee reported back the following officers of the Association for the present year:—

*Secretaries.*—Dr. H. A. Johnson, Ill.; Dr. Guido Furman, N. Y.

*Committee of Arrangements.*—Drs. James Anderson, N. Blakeman, T. M. Markoe, T. C. Finnell, Austin Flint, Jr., E. S. F. Arnold, J. H. Griscom.

*Committee on Prize Essays.*—Drs. D. F. Condie, Pa.; E. Wallace, Pa.; Wilson Jewell, Pa.; E. R. Peaslee, N. Y.; Alfred Stille, Pa.

*Committee on Medical Education.*—Drs. J. C. Dalton, N. Y.; M. L. Linton, Mo.; John Frissell, Va.; Howard Townsend, N. Y.; W. H. Byford, Ill.

*Committee on Medical Literature.*—Drs. L. M. Lawson, Ohio; D. L. McGugin, Iowa; William Mayberry, Pa.; H. Noble, Ill.; John Homans, Mass.

*Committee on Publication.*—Drs. F. G. Smith, Chairman, Pa.; Caspar Wistar, Pa.; Ed. Hartshorne, Pa.; H. F. Askew, Del.; S. G. Hubbard, Conn.; H. A. Johnson, Ill.; Guido Furman, N. Y.

*Committee on Insanity.*—Drs. Ralph Hill, Ohio; C. H. Nichols, D. C.; D. P. Bissell, N. Y.; S. W. Butler, Pa.; John S. Butler, Conn.

Dr. H. G. Davis commenced reading a paper on "The American Method of Treating Joint Diseases and Deformities," which was referred to the Committee of Publication, and its further reading suspended.

Dr. Homburger read a paper upon the use of the laryngiscope, exhibiting the instruments, and another upon a case of disappearance of the iris behind the lens. Referred to Committee of Publication.

The paper of Dr. Griscom, on a case of diarrhœa adiposa, (read on Thursday afternoon,) was, on motion of Dr. Furman, referred to the Committee of Publication.

Dr. A. Fisher read a paper on the use of the Sulphites of Lime and Soda in the treatment of hospital gangrene, phlibitis, erysipelas, and other zymotic diseases. On motion, the paper was referred to a committee of three, of which the author is chairman, to continue his investigations, and report next year.

Dr. Cox, of the Army, offered two resolutions,—one of thanks to the Citizens of Chicago, for their kindness and hospitality shown to the members of the Association during its sessions here, and another of thanks to the retiring Secretary, Dr. Hubbard, for his able and faithful services.

The Amendments to the Constitution of the Association, proposed at the last meeting, were called up, discussed, and rejected.

A complimentary resolution, thanking the President and Secretary for their services, was adopted.

The following gentlemen, on motion of the Committee of Arrangements, were elected permanent members of the Association:—

L. H. Cary, Toledo, Iowa; Horatio Hitchcock, Chicago; L. F. Warner, Chicago; L. P. Cheney, Chicago; C. W. Shumway, Chicago.

Adjourned till 3 P.M.

AFTERNOON SESSION.

The Convention assembled and was called to order by the President, at 3 o'clock. The minutes of the morning session were read and approved.

A letter was read from Dr. Russell, of Mt. Vernon, Ohio, asking to be excused from further service on a special committee. On motion, he was excused. A similar communication was also read from Prof. Sager, of Michigan, and disposed of in the same manner.

Dr. N. S. Davis offered an amendment to the Constitution, providing for the appointment of one permanent Secretary. Under the rules, the amendment lays over one year.

The Committee on Nominations reported the appointment of numerous gentlemen to act upon various matters that might come before the next annual meeting.

The following resolution was offered by Dr. Arnold, and passed:—

Whereas, The railroad is fast becoming the great medium of land travel in all parts of the world; and, whereas, in spite of all regulations and care, serious accidents are continually occurring, attended with loss of life, such being greatly augmented by the total want of any local medical provision to meet such, as well as by the absence of any appliances whatever, calculated to strengthen the hands of the surgeon; therefore, be it

*Resolved*, That such medical provision shall be made by the railroads; and that by the diminution of suffering, as well as by the saving of life, while economy will accure to the railroad companies, and the interests of humanity greatly served.

A lengthy memorial was received and read from the Special Committee appointed to address Congress in relation to the rank and pay of army-surgeons. On motion, the Report was accepted and adopted.

On motion, the Secretary was instructed to have the Memorial printed, and to send copies of the same to public officers at Washington.

After some further proceedings of an informal nature, the Convention adjourned.

